# Homing pigeon navigational ontogeny: no evidence that exposure to a novel release site is sufficient for learning

**DOI:** 10.1016/j.anbehav.2024.06.009

**Published:** 2024-08

**Authors:** Joe Morford, Anna Gagliardo, Enrica Pollonara, Tim Guilford

**Affiliations:** a Department of Biology, University of Oxford, Oxford, U.K.; b Department of Biology, University of Pisa, Pisa, Italy

**Keywords:** homing pigeon, learning, navigation, ontogeny

## Abstract

The navigational mechanisms of homing pigeons, *Columba livia*, have been extensively studied and represent a useful model for the navigation of birds and other animals. Pigeons navigate with an olfactory map and sun compass from unfamiliar areas and, in familiar areas, are largely guided by visual landscape cues, following stereotyped and idiosyncratic routes. However, the mechanisms by which they gain familiarity, improve their navigation and transition between navigational strategies during learning are not fully understood. Addressing these outstanding questions in this navigational model will help to improve our understanding of navigational ontogeny. We sought to investigate whether passive exposure to the cues at a site, without release, was sufficient for navigational learning, given that pigeons can determine the home direction before taking off. We exposed pigeons to cues at a novel site before returning them to the site the next day and releasing them alongside controls. We found no differences in the directional distributions, mean vector lengths, virtual vanishing times, efficiency indices or homing efficiency indices between birds that had and had not previously visited the site. We therefore found no evidence to suggest that passive exposure to the cues at a novel site was sufficient to facilitate a detectable improvement in navigational performance. There are three possible explanations for this result: first, a larger sample size would have detected a weak effect of learning; second, passive exposure to a release site is insufficient to generate navigational learning; and third, pigeons learn from passive exposure but do not rely upon this information, showing no difference in performance, despite learning. We discuss these three explanations with reference to previous findings on navigational learning in homing pigeons. We suggest that experiments should continue to examine navigational ontogeny in homing pigeons to help address this major problem for the field of navigation.

## Highlights

• The ways navigational learning proceeds in homing pigeons are not fully understood.
• We investigated whether exposure at a novel site is sufficient for learning.
• We found no evidence that exposure to a novel site improved homing performance.

Homing pigeons, *Columba livia*, represent an excellent model species to investigate questions relating to the navigation of birds and other animals. The mechanisms by which they navigate home in both familiar and unfamiliar areas have been extensively studied, and many of these findings have been highly applicable to animals across a range of contexts and taxa (Bonadonna & Gagliardo, 2021; Gagliardo et al., 2013; Padget et al., 2017, 2018; Perez et al., 1997; Pollonara et al., 2015; Safi et al., 2016; Wiltschko & Balda, 1989).

From unfamiliar areas, pigeons are able to navigate home through a ‘map-and-compass’ mechanism (Kramer, 1953), using a distributed map of environmental odour cues (Gagliardo, 2013; Gagliardo et al., 2011, 2018; Papi et al., 1972; Wallraff, 2005, 2014) and a time-compensated sun compass (Guilford & Taylor, 2014; Schmidt-Koenig, 1990). Learning of an olfactory map occurs during the first months after fledging, when pigeons memorize the odours carried by winds blowing at their home loft in association with the wind's direction (Gagliardo, Ioalè, et al., 2001; Odetti et al., 2003). Then, upon displacement, pigeons can determine their direction of displacement from the loft by relying on local environmental odour cues at the release site and can orient in that direction using their sun compass (Gagliardo, 2013).

Soon after release and during the entire homing flight, pigeons gain familiarity with the traversed areas, so that even after a single homing experience from a site, birds improve their navigational efficiency, with better homeward orientation and shorter flight paths (Gagliardo, Pollonara, & Wikelski, 2020). This improvement is likely driven by learning of both olfactory and visual features of the release site and subsequently overflown areas. With successive releases, they gradually develop fidelity to individual idiosyncratic routes (Biro et al., 2004; Guilford & Biro, 2014; Meade et al., 2005). This is presumably concomitant with a change in navigational strategy dominance from solely using distributed odour cues in combination with the sun compass when the site is initially unfamiliar to using visually guided mechanisms after both the site and the overflown landscape have become familiar.

A number of studies have shown that displaced pigeons are able to pick up navigational information before being released, and even make navigational decisions before taking off. For instance, exposure to local olfactory cues at an unfamiliar release site prior to release is necessary and sufficient for homeward orientation in pigeons released in anosmic condition (Gagliardo et al., 2016; Wallraff et al., 1984). Consistently, an Immediate Early Genes study on pigeons showed activation of the piriform cortex, an associative telencephalic region involved in olfactory navigation, after exposure to environmental cues at the release site, even if not released (Gagliardo et al., 1997; Papi & Casini, 1990; Patzke et al., 2010). This observation, together with experiments showing homeward orientation in a circular arena before take-off (Chelazzi & Pardi, 1972; Gagliardo, Odetti, et al., 2001; Mazzotto et al., 1999), suggests that pigeons start to process olfactory information before being released (Gagliardo, Odetti, et al., 2001). Additionally, exposure to the visual cues of a familiar release site before release can affect homing performance (Biro et al., 2003; Braithwaite, 1993; Braithwaite and Guilford, 1991, 1993; Braithwaite & Newman, 1994), allowing pigeons to determine the home direction before they take off (Gagliardo, Odetti, et al., 2001). These data raise the question of whether passive exposure to olfactory and visual cues at a novel release site might increase familiarity of pigeons to the site and facilitate navigational decisions, allowing faster vanishing times and lower uncertainty in taking an orientation decision upon subsequent release.

We designed an experiment to determine whether passive exposure to the environmental cues at a release site would be sufficient to improve navigational performance after release on a subsequent day. We exposed pigeons to the environmental cues, including visual and olfactory cues, at one of two sites, before returning them to their loft. On a subsequent day, the pigeons were released either at the same site (the experimental treatment group) or at the other release site (the controls). We predicted that if passive exposure to the cues at a site would facilitate some navigational learning or increase in familiarity with the site, the vanishing metrics (individual mean directions, mean vector lengths, virtual vanishing times, efficiency indices and homing efficiency indices) would differ between the two groups in the first kilometres from the release site.

## Methods

Forty-nine first-year pigeons hatched at the Arnino field station (43°39′26″N, 10°18′14″E), Pisa, Italy, were used in this study. The pigeons were housed in an aviary and had some experience free-flying and with local (<5 km) training releases. At least 2 weeks prior to the experimental releases, all the birds were equipped with a PVC dummy weight, similar in dimension and mass to the GPS data logger they would be carrying, to accustom them to flying with a load. The dummy was attached to the pigeons' back by means of a Velcro strip glued on the feathers, which had been trimmed. For releases, the pigeons were tracked with Mobile Action IgotU (20 g) GPS loggers, with a sampling rate of 1 Hz. The positional fixes stored by the loggers included latitude, longitude, date-time and speed.

On 10 August 2021, 12 pigeons were transported to a site in Castelfranco di Sotto (site 1; bearing home 261°; distance 34.2 km), and 12 to a site in Cecina (site 2; bearing home 336°; distance 39.3 km). At these sites, the pigeons were placed in cages on the roof of the car such that they could view the surrounding landscape. The cages had one open face and an open top and were turned through 90 degrees every half hour (three times). The pigeons were therefore exposed to the cues at the site for 2 h and facing in all directions. On the subsequent day, all 24 birds were transported to site 1 to be released. After arrival at the release site, the birds were kept inside an opaque airtight container ventilated by air that was filtered through an active charcoal filter. This removed most of the natural odorants contained in the air to ensure that those released last had not had much greater exposure to the olfactory cues at the release site than those released first. The birds were released individually, alternating between treatments, in sunny conditions, with light wind between 0835 and 1120 local time.

Similarly on 12 August, 12 pigeons were transported to site 1 and 13 to site 2 and exposed to the site for 2 h using the procedure described above. The next day, these pigeons were released from site 2 between 0835 and 1050.

A summary of the experimental design is shown in Table 1. Half of the 49 birds had been exposed to the cues at their release site (experimental treatment; in bold in Table 1), and half to the cues at a different, far-off release site (control treatment; in italics in Table 1).

**Table 1:** Experimental design and sample sizes

	Released at site 1	Released at site 2
Exposed at site 1	**12**	*12*
Exposed at site 2	*12*	**13**

Pigeons in the experimental group, which were exposed to a release site and then released from the same site on a subsequent day, are in bold; pigeons in the control group, which were exposed at one site and then released on a subsequent day at a different, far-off site, are in italics.

### Quantitative Analyses and Statistical Procedure

To exclude data likely to capture an initial escape response immediately following release, the initial fixes of the track within a 500 m radius of the release site were removed from all analyses. The initial sections of homing in which the pigeons left the release site were then analysed by isolating the section of the tracks before they left a 2 km radius of the release site for the final time. The track sections were visually inspected for following behaviours, in which the tracks of two or more birds closely followed each other for a period of time. Where following was observed, if the two or more birds flying together were in the same treatment group, one was randomly excluded from the analyses. However, if they were in different treatment groups, both were excluded, as it would not be possible to attribute any observed behaviours to either treatment group. Fixes with a recorded speed lower than 5 km/h were excluded from the analyses to remove stops.

We tested for differences between the groups in their directional distributions of the successive orientations of birds between leaving a 500 m radius of the release site and leaving a 2 km radius for the last time. This was tested using two-sample Hotelling's T-squared tests (Batschelet, 1981).

Additionally, for each track, four metrics were calculated: the virtual vanishing time, the individual mean vector length, the homing efficiency index and the efficiency index. The virtual vanishing time represents the time taken for the track to leave the 2 km radius of the release site for the final time. The mean vector length was taken from the calculated individual mean vector averaging the directions, with respect to home, taken by the bird while moving from one point to the next. The homing efficiency index quantifies the efficiency with which the track approaches home (Gagliardo et al., 2016). It is equal to the difference between the distance to home from the release site and the distance to home from where the track leaves the 2 km radius of the release site for the final time, divided by the path length. If the homing efficiency equals 1, the pigeon moved in a straight line from the release site in the direction of home. Finally, the efficiency index was calculated; this is the distance between first and last points in these track sections, divided by the path length.

Differences in these metrics between the treatment groups were assessed with ANOVA Type II linear models, with the general formula:

Metric ∼ Treatment group + Release site

These analyses were repeated with the section of the tracks before the pigeons leave a 5 km radius of the release site for the final time.

To test the assumption of normally distributed residuals, we visually inspected the quantile-quantile plots of the standardized residual quantiles against theoretical quantiles. To better fit this assumption, after visual inspection, the virtual vanishing times were log-transformed (natural logarithm). Additionally, we performed Shapiro – Wilk tests of normality on the residuals of each model. These tests found marginally significant (0.03 < *P* < 0.05) evidence of deviation from normality of the residuals in three of eight fitted linear models; there was no evidence that the remaining five models deviated from normality (*P* > 0.05). Given these marginally significant effects and the robustness of linear models to the relaxing of this assumption (Knief & Forstmeier, 2021), we considered linear models to be appropriate for this analysis.

We report *F* values, degrees of freedom, partial η^2^ (η^2^p) effect size values (Cohen, 1988), the fitted mean differences (with positive values signifying greater metric values in the experimental group than the treatment group, and in site 2 than site 1), along with the associated confidence intervals, Cohen's *d* standardized effect sizes (Cohen, 1988), along with associated confidence intervals, and *P* values.

Statistical analysis and graphical output was produced using R, using the R packages: circular (Agostinelli & Lund, 2022), geosphere (Hijmans, 2022), car (Fox & Weisberg, 2011), CircStats (Lund & Agostinelli, 2018), Hotelling (Curran & Hersh, 2021), scales (Wickham, 2018), useful (Lander, 2018), beeswarm (Eklund & Trimble, 2021), jmv (Selker et al., 2023) and pwrss (Bulus, 2023).

### Ethical Note

The pigeons were bred and manipulated in accordance with the 57 EU Directive 2010/63/EU on the protection of animals used for scientific purposes. The experiment was approved by the Scientific Ethics Committee of the University of Pisa and by the Italian Ministry of Health (permit number 238/2020-PR).

After any manipulation (application of the Velcro strip) the birds were released in their aviary or in front of it. The opportunity to fly freely around is thought to diminish the stress due to the manipulation. To accustom the pigeons to carrying a GPS device, the birds were fitted with PVC dummies for 2 weeks prior to the first release.

## Results

**Figure 1:**
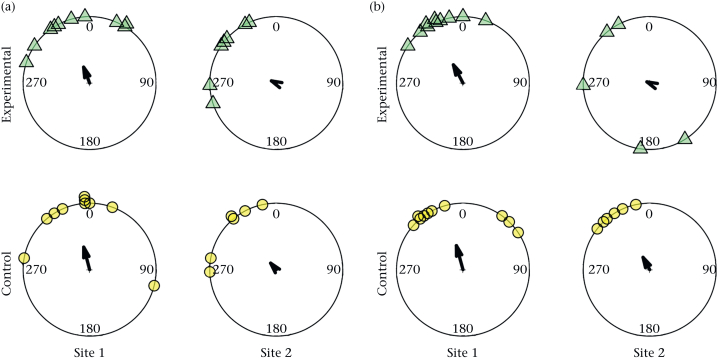
Directional distributions of initial orientations. The directions of the successive orientations of birds with respect to home (at 0 degrees), from release up to leaving (a) 2 km or (b) 5 km from the release site (site 1 or 2) for the final time. Each group is represented by a second-order mean vector of successive orientations. The mean direction of successive orientations of birds from the experimental group are represented by green triangles and the mean direction of birds from the control group are represented by yellow circles.

The directional distributions of the different groups are shown in Fig. 1. Each bird is represented by a mean direction of successive orientations with respect to home at 0 degrees. In all cases, the second-order mean vectors of successive orientations were directed anticlockwise of the home direction, with longer vectors (indicating stronger orientation) observed in groups of pigeons released at site 1 than site 2. No differences were found in the directional distributions of the two treatment groups after leaving a 2 km or 5 km radius of the release site (two-sample Hotelling's T-squared tests: 2 km radius: *N* = 33, test statistic = 1.5, *P* = 0.49; 5 km radius: *N* = 31, test statistic = 2.8, *P* = 0.27). There was a significant difference in the directional distributions of the groups released at the two different sites after leaving a 2 km radius of the release site and after leaving a 5 km radius of the release site (two-sample Hotelling's T-squared tests: 2 km radius: *N* = 33, test statistic = 11, *P* = 0.012; 5 km: *N* = 31, test statistic = 9.7, *P* = 0.018).

**Figure 2:**
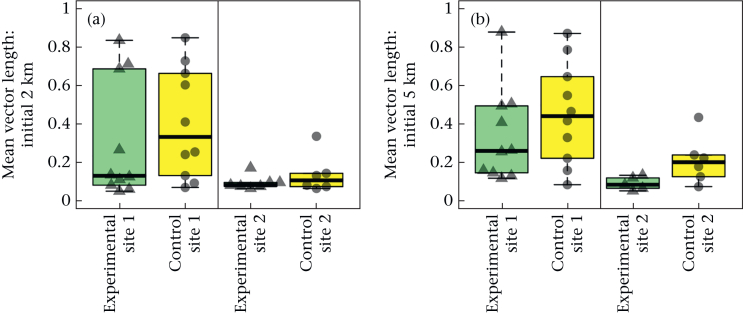
Mean vector lengths of successive orientations in initial homing paths. Box plots show the mean vector lengths of successive orientations of birds leaving (a) 2 km or (b) 5 km from the release site (site 1 or 2) for the final time. Boxes show the median and 25th and 75th percentiles; whiskers indicate values within 1.5 times the interquartile range. The data points of the mean vector lengths for the individual birds are also shown over the top of the box plots. The experimental group is represented by green box plots and triangular points, and the control group by yellow box plots and circular points.

We found no significant difference in the mean vector length of successive orientations (Fig. 2) at a 2 km or 5 km radius between the treatment groups (ANOVA Type II linear models: 2 km radius: *N* = 33, *F*_1,30_ = 0.87, η^2^p = 0.028, fitted mean difference = −0.076; confidence interval = (−0.24, 0.091), Cohen's *d* = −0.32, confidence interval = (−1.0, 0.39), *P* = 0.36; 5 km radius: *N* = 31, *F*_1,28_ = 2.5, η^2^p = 0.081, fitted mean difference = −0.12, confidence interval = (−0.27, 0.036), Cohen's *d* = −0.57, confidence interval = (−1.3, 0.19), *P* = 0.13). There was a strong effect of release site on the strength of orientation, with a significantly greater mean vector length at site 1 than at site 2 at both a 2 km and 5 km radius (2 km radius: *F*_1,30_ = 8.2, η^2^p = 0.22, fitted mean difference = −0.24, confidence interval = (−0.41, 0.069), Cohen's *d* = −1.0, confidence interval = (−1.8, −0.25), *P* = 0.008; 5 km radius: *F*_1,28_ = 9.5, η^2^p = 0.25, fitted mean difference = −0.24, confidence interval = (−0.40, −0.081), Cohen's *d* = −1.2, confidence interval = (−2.0, −0.33), *P* = 0.005), indicating a stronger orientation of birds released at site 1 than site 2.

**Figure 3:**
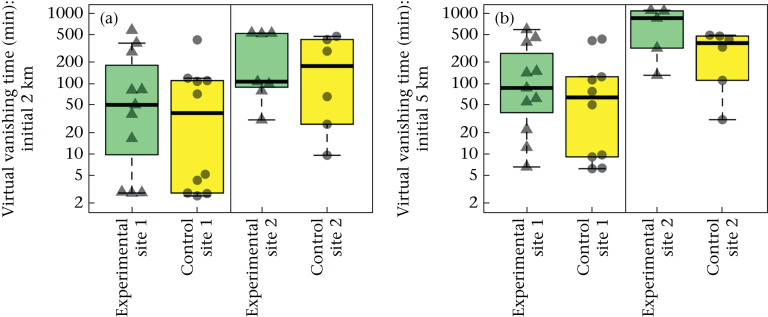
Virtual vanishing times. Box plots show the virtual vanishing time (min) of birds leaving (a) 2 km or (b) 5 km from the release site (site 1 or 2) for the final time. Boxes show the median and 25th and 75th percentiles; whiskers indicate values within 1.5 times the interquartile range. The data points of the virtual vanishing times for the individual birds are also shown over the top of the box plots. The experimental group is represented by green box plots and triangular points, and the control group by yellow box plots and circular points. The virtual vanishing time is shown on a logarithmic scale.

We found no significant difference in the virtual vanishing time (log-transformed) of the tracks (Fig. 3) at a 2 km or 5 km radius between the treatment groups (ANOVA Type II linear models: 2 km radius: *N* = 34, *F*_1,31_ = 0.89, η^2^p = 0.028, fitted mean difference = 0.57, confidence interval = (−0.66, 1.80), Cohen's *d* = 0.32, confidence interval = (−0.38, 1.0), *P* = 0.35; 5 km radius: *N* = 32, *F*_1,29_ = 2.0, η^2^p = 0.065, fitted mean difference = 0.70, confidence interval = (−0.31, 1.71), Cohen's *d* = 0.50, confidence interval = (−0.23, 1.2), *P* = 0.17). There was a strong effect of release site on the virtual vanishing time, with significantly greater virtual vanishing time in the birds released at site 2 than at site 1 at a 2 km radius (*F*_1,31_ = 5.8, η^2^p = 0.16, fitted mean difference = 1.5, confidence interval = (0.22, 2.7), Cohen's *d* = 0.85, confidence interval = (0.095, 1.6), *P* = 0.022) and a 5 km radius (*F*_1,29_ = 11, η^2^p = 0.28, fitted mean difference = 1.7, confidence interval = (0.66, 2.8), Cohen's *d* = 1.2, confidence interval = (0.41, 2.1), *P* = 0.002), indicating that birds released at site 1 were quicker to leave the vicinity of the release site than those released at site 2.

**Figure 4:**
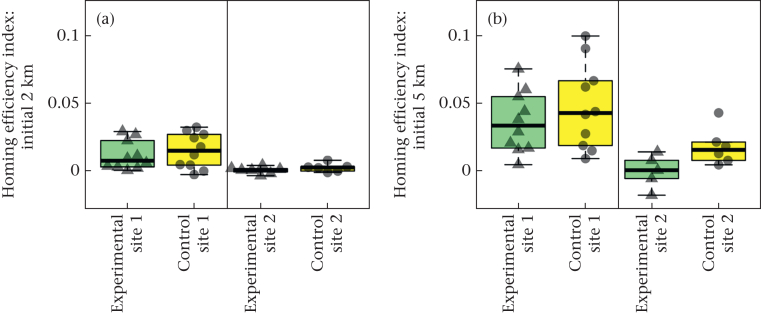
Initial homing efficiencies. Box plots show the homing efficiency index of birds leaving (a) 2 km or (b) 5 km from the release site (site 1 or 2) for the final time. Boxes show the median and 25th and 75th percentiles; whiskers indicate values within 1.5 times the interquartile range. The data points for the individual birds are also shown over the top of the box plots. The experimental group is represented by green box plots and triangular points, and the control group by yellow box plots and circular points.

We found no significant difference in the homing efficiency index (Fig. 4) of the tracks at a 2 km or 5 km radius between the treatment groups (ANOVA Type II linear models: 2 km radius: *N* = 33, *F*_1,30_ = 0.81, η^2^p = 0.026, fitted mean difference = −0.0029, confidence interval = (−0.0096, 0.0037), Cohen's *d* = −0.31, confidence interval = (−1.0, 0.40), *P* = 0.37; 5 km: *N* = 31, *F*_1,28_ = 2.7, η^2^p = 0.089, fitted mean difference = −0.014, confidence interval = (−0.031, 0.0033), Cohen's *d* = −0.60, confidence interval = (−1.4, 0.16), *P* = 0.11). There was a strong effect of release site on homing efficiency, with a significantly greater homing efficiency index at site 1 than at site 2 at both a 2 km and 5 km radius (2 km radius: *F*_1,30_ = 12, η^2^p = 0.29, fitted mean difference = −0.012, confidence interval = (−0.019, −0.0050), Cohen's *d* = −1.26, confidence interval = (−2.1, −0.46), *P* = 0.001; 5 km radius: *F*_1,28_ = 14, η^2^p = 0.34, fitted mean difference = −0.033, confidence interval = (−0.051, −0.015), Cohen's *d* = −1.4, confidence interval = (−2.3, −0.55), *P* = 0.001), indicating that birds released at site 1 initially approached home more efficiently than those released at site 2.

**Figure 5:**
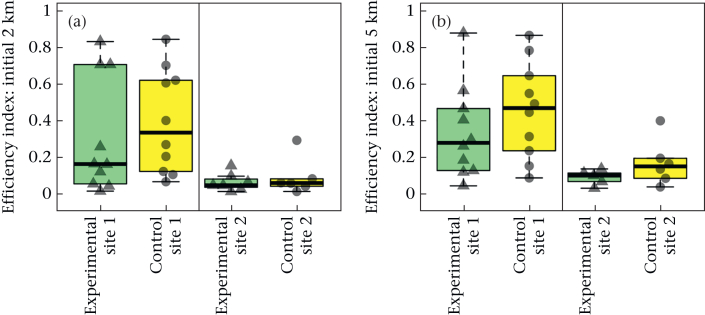
Initial efficiencies. Box plots show the efficiency index of birds leaving (a) 2 km or (b) 5 km from the release site (site 1 or 2) for the final time. Boxes show the median and 25th and 75th percentiles; whiskers indicate values within 1.5 times the interquartile range. The data points for the individual birds are also shown over the top of the box plots. The experimental group is represented by green box plots and triangular points, and the control group by yellow box plots and circular points.

Finally, we found no significant difference in the efficiency index of the tracks (Fig. 5) at a 2 km or 5 km radius between the treatment groups (ANOVA Type II linear models: 2 km radius: *N* = 33, *F*_1,30_ = 0.61, η^2^p = 0.020, fitted mean difference = −0.064, confidence interval = (−0.23, 0.10), Cohen's *d* = −0.27, confidence interval = (−0.99, 0.44), *P* = 0.44; 5 km: *N* = 31, *F*_1,28_ = 1.9, η^2^p = 0.065, fitted mean difference  = −0.11, confidence interval = (−0.26, 0.050), Cohen's *d* = −0.50, confidence interval = (−1.3, 0.25), *P* = 0.17). There was a strong effect of release site on efficiency, with a significantly greater efficiency index at site 1 than at site 2 at both a 2 km and 5 km radius (2 km radius: *F*_1,30_ = 10, η^2^p = 0.26, fitted mean difference = −0.27, confidence interval = (−0.44, −0.10), Cohen's *d* = −1.2, confidence interval = (−1.9, −0.36), *P* = 0.003; 5 km radius: *F*_1,28_ = 11, η^2^ = 0.29, fitted mean difference = −0.27, confidence interval = (−0.43, −0.10), Cohen's *d* = −1.3, confidence interval = (−2.1, −0.42), *P* = 0.002), indicating that birds released at site 1 had more efficient initial trajectories than those released at site 2.

Despite the, in principle, problems with carrying out post hoc power analyses to aid the interpretation of nonsignificant results (Dziak et al., 2020; Heckman et al., 2022), here we report the statistical power, β, associated with testing for an effect of treatment in these linear models. Power is reported for four baseline η^2^ effect sizes: (1) the mean η^2^p effect size of release site, 0.26, found in the eight models given above, representing a realistic baseline expectation of the size of an effect that we might have expected to find in relation to treatment in this experiment, and three conventional ranges for (2) strong, η^2^p > 0.14, (3) medium, 0.06 < η^2^p < 0.14 and (4) weak, 0.01 < η^2^p < 0.06, η^2^p effect sizes (Cohen, 1988). The results of the power analysis are presented in Table 2, for both the smallest (*N* = 31) and largest (*N* = 34) sample sizes of the analyses presented above.

**Table 2:** Power analysis

	Partial η^2^ effect size	Power, β, with *N* = 31 (%)	Power, β, with *N* = 34 (%)
Average effect size of release site	η^2^p = 0.26	β = 88.9	β = 91.7
Strong	η^2^p > 0.14	β > 58.3	β > 62.5
Medium	0.06 < η^2^p < 0.14	27.4 < β < 58.3	29.8 < β < 62.5
Weak	0.01 < η^2^p < 0.06	8.4 < β < 27.4	8.8 < β < 29.8

Statistical power is presented for a range of baseline effect sizes (partial η^2^) and two sample sizes (the smallest and largest of the analyses presented here).

## Discussion

Overall, we found no differences in the initial homing performance of pigeons that had previously been passively exposed to a release site compared to those that had not, but a range of significant differences in the initial homing performance of pigeons released at different release sites. Release site-specific effects on the initial homing performance of pigeons are well established (often termed release site bias) and are expected to play a larger role at unfamiliar release sites (Keeton, 1973; Kowalski & Wiltschko, 1987; Kramer, 1957; Wallraff, 1959). We found differences between release sites in the directional distribution of initial orientation and found that pigeons at site 1 left the vicinity of the release site more quickly and had a greater strength of orientation (mean vector length), a greater efficiency and a greater efficiency of approaching home than pigeons released at site 2. Conversely, we found no differences between treatment groups in their directional distributions, their time taken to leave the release site, their initial strength of orientation, their efficiency or their efficiency of approaching home. Hence, we found no evidence to suggest that passive exposure to a release site was sufficient to generate any improvement in navigational performance. There are three possible explanations for this result: the first is that the sample size of the experiment was not large enough to detect a weak effect, but that it might have been detected with a larger sample size; the second is that passive exposure to a release site is insufficient to generate navigational learning, and hence pigeons cannot or do not learn from passive exposure to a novel site; the third is that pigeons learn from passive exposure to the release site, but do not rely upon what they have learned, showing no difference in performance, despite learning.

The first explanation for the lack of any detectable navigational learning generated by passive exposure to a release site is that some navigational learning does occur, but our experiment was unable to detect it. This would be possible if the effect of learning after passive exposure to the release site were weak, especially given that some data had to be excluded from our analyses when pigeons followed each other after release. This left, in the analyses where the greatest number of birds had to be excluded, 15 in the experimental group and 16 in the control group, split across the two release sites. Nevertheless, our power analysis indicates that we had a high probability (at least 88% in all reported analyses) of detecting realistic possible effects of treatment (an effect size of the same magnitude as the average effect of release site in this experiment). Additionally, we had reasonably high statistical power (at least 58% in all reported analyses) to detect ‘strong’ effects of treatment, and reasonable power to detect ‘moderate’ effects of treatment (between 27% and 63% in all reported analyses), as have been conventionally defined (Cohen, 1988). Additionally, one of the most directly comparable available studies (Gagliardo, Pollonara, & Wikelski, 2020) found mean increases of 0.15, 0.26 and 0.22 in the efficiency index of (control) pigeons after a single release at three sites. Each of these increases was greater than our estimated upper bound of the confidence intervals for the improvements in efficiency indices (2 km: 0.10; 5 km: 0.050) and homing efficiency indices (2 km: 0.0037; 5 km: 0.0033) after passive exposure to the release site in this study (equivalent to fitted mean difference between treatments here). This indicates that any increase in efficiency generated by passive exposure to a release site in this experiment was less than the reported increases in efficiency generated by a single previous release in a similar experiment. Further experiments could include both passive exposure and single release at a novel release site as different treatments in the same experiment to directly compare the effect of these treatments on learning and subsequent homing. Hence, it is unlikely that a large effect of passive exposure, equivalent to the effect of a previous release at the site, on the subsequent homing of pigeons would not have been detected by this experiment. However, a weak effect of treatment and an insufficiently large sample size could potentially explain the lack of any detectable navigational learning in this experiment.

A second possibility is that passive exposure to a release site is insufficient for navigational learning. Previous experiments have shown that pigeons are able to determine the home direction at release sites before take-off (Chelazzi & Pardi, 1972; Gagliardo, Odetti, et al., 2001; Mazzotto et al., 1999), and that passive exposure to a release site activates a greater number of neurons in the piriform cortex than release in the vicinity of home (Patzke et al., 2010). However, neurons in the hippocampal formation are activated significantly less in pigeons after passive exposure to release sites than birds released and able to home from the same sites (Patzke et al., 2010). This is another important brain region for spatial learning in homing pigeons (Bingman et al., 2005), involved in developing route-following mechanisms from familiar sites, although not in developing site-specific compass orientation (Gagliardo et al., 1999; Gagliardo et al., 2002, 2009; Gagliardo, Pollonara, Casini, et al., 2020). Similar results were recently reported for young domestic chicks, *Gallus gallus domesticus*, showing that hippocampal activation was significantly higher in chicks that could explore a novel environment, than in chicks that could only observe the same space without exploring it (Morandi-Raikova & Mayer, 2022). These Immediate Early Gene activation findings therefore fit with this explanation of our results that passive exposure to the cues of a release site may be insufficient to generate navigational learning, possibly because it fails to activate sufficiently their hippocampal formation and trigger learning about the visual cues at the site.

Therefore, one could hypothesize that flight, exploration and/or homing at a novel release site might be necessary for learning, whereas passive exposure is insufficient. This could explain why we found no effect of a single passive exposure to a site, despite a previous experiment showing that a single release from an unfamiliar site is sufficient for navigational learning using visual cues (Gagliardo, Pollonara, & Wikelski, 2020), with these cues relied upon more by pigeons subsequently released after made anosmic than by controls. However, the difference between anosmic and control pigeons in that experiment was observed in the ‘en route navigation’ rather than ‘initial decision making’ of the pigeons. It is therefore also unclear how much pigeons learn about the visual cues at a release site after a single release.

On the other hand, homing pigeons appear able to remember olfactory information after passive exposure to a novel release site for at least a few hours, as shown by ‘site simulation experiments’ (Benvenuti & Wallraff, 1985; Dall'Antonia et al., 1999). In these experiments, birds displayed orientations consistent with the home direction from the site where they had been exposed to environmental odours a few hours before. It is possible that exposure to the olfactory cues at a site in these experiments was sufficient for pigeons to make an orientation decision which is retained for some hours but would not be retained until a subsequent day, and hence not constitute navigational learning or an increase in familiarity with the site.

Finally, a third possible explanation of our results is that pigeons learn through passive exposure to a new release site, but none the less show no difference in subsequent navigational performance. This possibility is also demonstrated by the difference in reliance on visual cues after a single release from a site between pigeons released subsequently either anosmic or intact (Gagliardo, Pollonara, & Wikelski, 2020). This experiment demonstrates that pigeons can learn about various cues after release at a new site but rely upon this information to different extents when subsequently released in different conditions (anosmic or intact), and hence that the transition between navigational strategies may not be limited by the rate of learning of visual landmarks. This highlights the distinction between learning and performance and opens the possibility that the pigeons in our experiment might have learned about the cues at the release site, but not relied upon the information they had learned when subsequently released.

In summary, we found no evidence of an increase in navigational performance after exposure to the cues at a novel site without release or homing from the site. This suggests that any effect of learning generated by passive exposure to sites may be weak or not reflected in navigational performance upon subsequent release. We believe that further experiments should continue to probe outstanding questions relating to navigational learning and ontogeny in homing pigeons and other animals, to better address this still outstanding major problem of ontogeny for the field of navigation, and ethology more generally (Tinbergen, 1963).

## Author Contributions

**Joe Morford:** Writing – review & editing, Writing – original draft, Visualization, Methodology, Investigation, Formal analysis, Data curation, Conceptualization. **Anna Gagliardo:** Writing – review & editing, Supervision, Methodology, Investigation, Conceptualization. **Enrica Pollonara:** Methodology. **Tim Guilford:** Writing – review & editing, Supervision, Conceptualization.

## Data Availability

The data supporting the conclusions of this article can be accessed on GitHub: https://github.com/Morfordjoe/Pigeon-Navigational-Learning-Pisa-2021

## Declaration of Interest

The authors have no conflicts of interest.
